# View on the bullishness index and agreement index

**DOI:** 10.3389/fpsyg.2022.957323

**Published:** 2022-07-28

**Authors:** Qing Liu, Xueqing Zhou, Lilu Zhao

**Affiliations:** ^1^College of Economics and Management, Huainan Normal University, Huainan, China; ^2^Graduate School of Management of Technology, Pukyong National University, Busan, South Korea; ^3^Communist Youth League, Huainan Normal University, Huainan, China; ^4^School of Mathematics, Shandong University, Jinan, China

**Keywords:** bullishness index, agreement index, comment, mathematical description, data simulation

## Introduction

Antweiler and Frank's (2004) paper “Is All That Talk Just Noise? The Information Content of Internet Stock Message Boards” published in The Journal of Finance, is an important piece of literature on the relationship between investor sentiment and stock markets based on natural language processing. More than 2,400 research publications have acknowledged its techniques, background, or conclusions (Google Scholar, 2022.5.17), making it an outstanding academic resource for future researchers. This article is not intended to challenge the paper's interpretation or conclusions, but rather to provide further references to the “bullishness index” and “agreement index.”

Antweiler and Frank denote the number of bullish, bearish, and neutral messages in a period *t* using the symbols $M_{t}^{BUY},$ $M_{t}^{SELL}$, and $M_{t}^{HOLD}$, respectively. After aggregating the messages, they created three functions to calculate the bullish signal, using the formula


(1) $$\begin{array}{l} M_{t}=M_{t}^{BUY}+M_{t}^{SELL} \end{array}$$

to represent the sum of messages related to the sentiment metric. The one used in the paper's report is the second function formula,


(2) $$\begin{array}{l} B_{t}^{{}^{*}}=ln\left[\frac{1+M_{t}^{BUY}}{1+M_{t}^{SELL}}\right]. \end{array}$$

Antweiler and Frank also created the “agreement index” to measure investor disagreement, which is calculated as follows:


(3) $$\begin{array}{l} A_{t}=1-\sqrt{1-{\left(\frac{M_{t}^{BUY}-M_{t}^{SELL}}{M_{t}^{BUY}+M_{t}^{SELL}}\right)}^{2}} \end{array}$$

In a recent study, we used the above formula to create a bullishness index and an agreement index to measure the volatility cycle of investor sentiment on social media. While visualizing the data, we discovered that the distribution of the “bullishness index” and “agreement index” is so neat that we find it difficult to believe that this is due to the data itself. As a result, we suspect that there is a strong relationship between Equations (2) and (3). We confirmed our suspicions with mathematical proof and data simulation. We discover that this association is absolute at larger, *M*_t_., regardless of the data examined in the study. When using the above formula to study investor sentiment, if a variable is correlated with the “bullishness index,” it is inevitably correlated with the “agreement index.” This is due to the formula, not the object under study. We're not sure if this is in conflict with the original intent of creating and analyzing the “bullishness index” and “agreement index,” respectively.

Traditional approaches to sentiment proxies are divided into three categories: market indices, survey indices, and special events. Market indicator proxies measure investor sentiment indirectly by using market indicators such as trading volume, closed-end fund discounts, first-day returns on initial public offerings (IPOs), and so on. Survey index-based proxies quantify investor sentiment by collecting optimistic or pessimistic expectations regarding the stock market from investors *via* surveys, such as the Consumer Confidence Index (Brown and Cliff, 2005), the Business Confidence Index (Liston-Perez et al., 2018), and the UBS/GALLUP Investor Optimism Index (Lemmon and Portniaguina, 2006). In a special event-based approach, special social events are frequently used as emotional proxies, and COVID-19 may be the most illustrative current example. Naseem et al. (2021) look at how COVID-19 affects the minds of investors and how that affects the stock market. Market-based measures have the advantage of being readily available at a relatively high frequency, but they also have the disadvantage of being the equilibrium result of many economic forces other than investor sentiment. The data acquisition of the proxy approach based on market indices is very slow, and it is usually done monthly or quarterly. The agent approach based on special things, on the other hand, is often used to analyze the impact of special events and is not universal.

“Google search queries” based investor sentiment proxies are easy to obtain and highly credible (Da et al., 2015), and are gaining more attention from scholars (Trichilli et al., 2018, 2020a,b,c). In addition to the emotional proxy methods mentioned above, natural language processing (NLP) technologies provide new ways to measure how investors feel. NLP is a research basis for social media-based investor sentiment research because it can tap into investor sentiment embedded in text and social networks. It also has the advantages of easy data availability, real-time access, and high credibility. It also gives us the chance to predict stock market returns based on high-frequency sentiment (Renault, 2017; McGurk et al., 2020). The research conducted by Antweiler and Frank (2004) was one of the very first to investigate the relationship between investor sentiment and the stock market based on social media. Researchers rely on their “bullishness index” and “agreement index” to construct investor sentiment (Rao and Srivastava, 2014; Checkley et al., 2017; Liu et al., 2017; Chernozhukov et al., 2018; Fallahgoul, 2021). These studies typically measure daily investor sentiment, resulting in a large *M*_*t*_ in formula (1) and a high correlation between the “bullishness index” and the “agreement index”. Xiong et al. analyzed the correlation between investor sentiment and the stock market. According to the paper's disclosures, the average daily total number of messages in that study was 10,443, and the average *M*_*t*_ was ~3,550 (Xiong et al., 2017).

In this age of exploding social media, natural language processing-based approaches to sentiment agents are growing in importance. Therefore, the research of Antweiler and Frank is increasingly cited by scholars. Due to the strong correlation between Equations (2) and (3), however, some of the researchers' conclusions regarding investor sentiment may be susceptible to systematic errors. They may not be able to confirm whether these conclusions are caused by the characteristics of the study subjects or by the formulas themselves. Disclosure of the relationship between the characteristics of the “bullishness index” and the “agreement index” will assist researchers in better constructing the investor sentiment index, thereby preventing analytical misunderstandings. This is essential for analyzing investor sentiment, disagreement, and their relationship.

In the subsequent section of the paper, we use mathematical functions and data simulation to describe the relationship between Equations (2) and (3).

## Mathematical description

To improve the readability of the paper, x is used below to denote the “bullishness index” $B_{t}^{*}$ and y is used to denote the “agreement index” *A*_*t*_.

From Equation (2)


$$\begin{array}{l} x=ln\left[\frac{1+M_{t}^{BUY}}{1+M_{t}^{SELL}}\right] \end{array}$$

we can get


$$\begin{array}{l} e^{x}\left(1+M_{t}^{SELL}\right)=1+M_{t}^{BUY}. \end{array}$$

Substituting Equation (1) into the above formula, we can get


$$\begin{array}{l} e^{x}+e^{x}M_{t}-e^{x}M_{t}^{BUY}=1+M_{t}^{BUY}, \end{array}$$

then


(4) $$\begin{array}{l} M_{t}^{BUY}=\frac{e^{x}+e^{x}M_{t}-1}{e^{x}+1}=\frac{e^{x}+\left(e^{x}+1\right)M_{t}-M_{t}-1}{e^{x}+1}=M_{t}-\frac{-e^{x}+M_{t}+1}{e^{x}+1}, \end{array}$$

and


(5) $$\begin{array}{l} M_{t}^{SELL}=M_{t}-M_{t}^{BUY}=\frac{-e^{x}+M_{t}+1}{e^{x}+1}. \end{array}$$

Combining Equations (4) and (5), we can get


(6) $$\begin{array}{l} M_{t}^{BUY}-M_{t}^{SELL}=M_{t}-\frac{-2e^{B_{t}}+2M_{t}+2}{e^{B_{t}}+1}\\ =\frac{e^{B_{t}}M_{t}+2e^{B_{t}}-2-M_{t}}{e^{B_{t}}+1}=\frac{\left(e^{B_{t}}-1\right)\left(M_{t}+2\right)}{e^{B_{t}}+1}. \end{array}$$

In Equation (3), introducing Equations (6) and (1) can obtain


$$\begin{array}{l} y=1-\sqrt{1-{\left(\frac{M_{t}^{BUY}-M_{t}^{SELL}}{M_{t}^{BUY}+M_{t}^{SELL}}\right)}^{2}}\\ \;=1-\sqrt{1-{\left(\frac{\left(e^{x}-1\right)\left(M_{t}+2\right)}{\left(e^{x}+1\right)M_{t}}\right)}^{2}}\\ \;=1-\sqrt{1-{\left(\frac{e^{x}-1}{e^{x}+1}\;\times \;\frac{M_{t}+2}{M_{t}}\right)}^{2}}, \end{array}$$

and approximate formula


(7) $$\begin{array}{l} y\approx 1-\frac{2e^{\frac{x}{2}}}{e^{x}+1}. \end{array}$$

## Data simulation

By controlling the total number of messages, N_t_ in period t, we indirectly control the number of valid data, M_t_. With data simulation, you can see how the “bullishness index” and the “agreement index” are related to each other.

Let the minimum value of investor message volume be 3 and the maximum value be N in this experiment. The message volume *N*_*t*_ of period t is a random number in the range [3, N], $M_{t}^{BUY}$ is a random number in the range [0, *N*_*t*_), and $M_{t}^{SELL}$ is a random number in the range [0, $N_{t}-M_{t}^{BUY}$]. In each experiment, 1095 simulated samples are made, and the samples are numbered from 0 to 1094. If (*x*_*t*_, *y*_*t*_) stands for the “bullishness index” and “agreement index” of period t, and *x*_*t*_ and *y*_*t*_ are found by using Equations (2) and (3), then the set of all sample points is defined as


$$\begin{array}{l} \left(X,Y\right)=\overset{1094}{\underset{t=0}{⋃}}\left(x_{t},y_{t}\right). \end{array}$$

**Figure 1:**
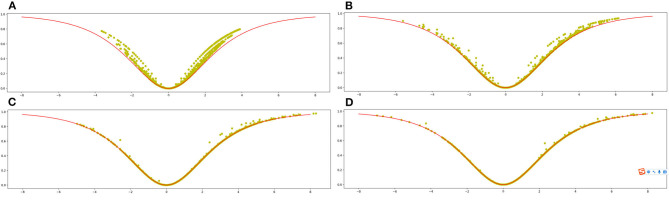
The relationship between the “bullishness index” and the “agreement index” in a simulation. **(A)** The simulation for *N* = 100, while **(B)** represents the simulation for *N* = 1,000. **(C)** The simulation for *N* = 10,000, while **(D)** represents the simulation for *N* = 20,000.

Figure 1A shows the scatter plot of the sample set (X, Y) when the control variable N is equal to 100. The black curve in Figure 1A is the plot of the approximate function


$$\begin{array}{l} y=1-\frac{2e^{\frac{x}{2}}}{e^{x}+1}. \end{array}$$

The ratio of *M*_*t*_+2 to *M*_*t*_ is relatively large when the number of messages in time period t is small, as shown in Figure 1A, and most random sample points are above the approximation curve. The shape of the sample distribution, on the other hand, is consistent with the approximation curve, and the “bullishness index” and the “agreement index” show statistical correlation.

Except for *N* = 1,000, the settings in Figure 1B are identical to those in Figure 1A. The random sample points fit the approximation curve better as the volume of investor messages increases in period t, and the correlation between the “bullishness index” and the “agreement index” increases.

The simulated graphs for *N* = 10,000 and *N* = 20,000 are shown in Figures 1C,D, where the relationship between the “bullishness index” and the “agreement index” is well fitted to the curve of Equation (7). With a very small error, the value of *y*_*t*_ can be deduced from x t using Equation (7) at this point.

In conclusion, the “bullishness index” created by Antweiler and Frank demonstrates a strong correlation with the “agreement index” even when the total sample size is small, indicating a statistical association.

## Conclusion

In the age of artificial intelligence and big data, the sentiment agent approach based on natural language processing is gaining importance (Farzindar and Inkpen, 2015). At the same time, sentiment measurement based on natural language processing has become an essential tool for governments, research institutions, and financial institutions to formulate industry policies and manage financial risks (Ku et al., 2008; Fisher et al., 2016). The “bullishness index” and “agreement index” developed by Antweiler and Frank are becoming increasingly cited by academics. In this paper, we use a mathematical formula to approximate the relationship between the “bullishness index” and the “agreement index” and examine the effect of the range of the total sample on the error of the formula through data simulation. Due to the correlation, the correlation analysis of investor sentiment and investor opinion disagreement using Equations (2) and (3) may produce conclusions that are unrelated to the underlying data. Although we do not consider the paper's conclusion, “Is All That Talk Just Noise? The Information Content of Internet Stock Message Boards” to be problematic, we advise avoiding the use of both Equations (2) and (3). Disclosure of the approximate relationship between the “bullishness index” and the “agreement index” is crucial for the study of investor sentiment, disagreement, and behavioral finance.

This paper demonstrates and confirms the relationship between the “bullishness index” and the “agreement index” and the possibility of analytical error. However, we do not offer a superior alternative. In future studies, we will propose potential substitutes.

## Author contributions

All authors listed have made a substantial, direct, and intellectual contribution to the work and approved it for publication.

## Conflict of interest

The authors declare that the research was conducted in the absence of any commercial or financial relationships that could be construed as a potential conflict of interest.

## Publisher's note

All claims expressed in this article are solely those of the authors and do not necessarily represent those of their affiliated organizations, or those of the publisher, the editors and the reviewers. Any product that may be evaluated in this article, or claim that may be made by its manufacturer, is not guaranteed or endorsed by the publisher.
